# Environment-Sensitive Fluorescence of 7-Nitrobenz-2-oxa-1,3-diazol-4-yl (NBD)-Labeled Ligands for Serotonin Receptors

**DOI:** 10.3390/molecules26133848

**Published:** 2021-06-24

**Authors:** Parijat Sarkar, Kaleeckal G. Harikumar, Satinder S. Rawat, Sanjib Das, Tushar K. Chakraborty, Amitabha Chattopadhyay

**Affiliations:** 1 CSIR-Centre for Cellular & Molecular Biology, Uppal Road, Hyderabad 500 007, India; parijat@ccmb.res.in (P.S.); harikumarkg@mayo.edu (K.G.H.); satinderrawat10@gmail.com (S.S.R.); 2 CSIR-Indian Institute of Chemical Technology, Uppal Road, Hyderabad 500 007, India; sanjib.das@glenmarkpharma.com (S.D.); tushar@iisc.ac.in (T.K.C.)

**Keywords:** NBD, serotonin, serotonin_1A_ receptor, confocal microscopy, spectral imaging, REES

## Abstract

Serotonin is a neurotransmitter that plays a crucial role in the regulation of several behavioral and cognitive functions by binding to a number of different serotonin receptors present on the cell surface. We report here the synthesis and characterization of several novel fluorescent analogs of serotonin in which the fluorescent NBD (7-nitrobenz-2-oxa-1,3-diazol-4-yl) group is covalently attached to serotonin. The fluorescent ligands compete with the serotonin_1A_ receptor specific radiolabeled agonist for binding to the receptor. Interestingly, these fluorescent ligands display a high environmental sensitivity of their fluorescence. Importantly, the human serotonin_1A_ receptor stably expressed in CHO-K1 cells could be specifically labeled with one of the fluorescent ligands with minimal nonspecific labeling. Interestingly, we show by spectral imaging that the NBD-labeled ligand exhibits a red edge excitation shift (REES) of 29 nm when bound to the receptor, implying that it is localized in a restricted microenvironment. Taken together, our results show that NBD-labeled serotonin analogs offer an attractive fluorescent approach for elucidating the molecular environment of the serotonin binding site in serotonin receptors. In view of the multiple roles played by the serotonergic systems in the central and peripheral nervous systems, these fluorescent ligands would be useful in future studies involving serotonin receptors.

## 1. Introduction

Serotonin (5-hydroxytryptamine) is a biogenic amine, rather serendipitously discovered ~70 years back [1], that acts as a neurotransmitter. Serotonin is localized in diverse regions both in the central and peripheral nervous systems [2]. It is found in organisms that span a wide evolutionary range, from humans to species with primitive nervous systems such as worms [3,4], and mediates a variety of physiological responses in distinct cell types. Signaling mediated by serotonergic systems plays a crucial role in the initiation and regulation of several behavioral and cognitive functions that include sleep, pain, depression, sexual activity, alcohol abuse, and learning [5,6,7,8,9,10]. Dysfunctional serotonergic signaling has been implicated in the etiology of mental disorders such as schizophrenia, depression, suicidal behavior, infantile autism, and obsessive–compulsive disorder [11,12,13]. Signaling via serotonin is mediated by binding of serotonin to specific receptors on the cell surface, which are pharmacologically categorized into many groups [4,14,15,16,17]. A majority of serotonin receptors belong to the superfamily of seven transmembrane domain G protein-coupled receptors (GPCRs) [4,16,18]. Amongst the members of the serotonin receptor family, the serotonin_1A_ receptor subtype is the most extensively studied GPCR for a variety of reasons [15,19,20,21].

We previously showed that the intrinsic fluorescence properties of serotonin are dependent on its ionization state [22]. Our results showed that the fluorescence of native serotonin (as monitored by intensity, emission maximum, and fluorescence lifetime) is pH-dependent. Further, since the ligand binding site for serotonin receptors is localized in the transmembrane core of the receptor, we monitored serotonin fluorescence in various nonpolar media in order to investigate the possibility of monitoring ligand binding in a sensitive and non-invasive way using the native fluorescence of serotonin. However, we found that the native fluorescence of serotonin is not ideally suited for this purpose due to a number of reasons. First, serotonin fluorescence was not found to be solvatochromic, i.e., not sensitive to its environment (as opposed to its parent fluorophore tryptophan, which shows large degree of solvatochromism). As a result, when the polarity of the environment in which serotonin was dissolved was changed, the corresponding changes in various fluorescence parameters were minimal or negligible. An additional problem arises due to the fact that serotonin absorbs and emits in the UV region of the spectrum. This makes serotonin fluorescence unsuitable for microscopy-based applications, since the sensitivities of most detectors for fluorescence microscopy fall off in the UV region. More importantly, many optical components of the microscope, unless specifically designed for UV transmission, absorb strongly in this wavelength range [23]. In addition, serotonin fluorescence has considerable overlap with the tryptophan fluorescence emitted by proteins, which could give rise to additional complications.

An extensively used fluorophore in biochemical, biophysical, and cell biological studies is the NBD (7-nitrobenz-2-oxa-1,3-diazol-4-yl) group [24,25]. The NBD group fluoresces in the visible region, and the excitation wavelength is compatible for excitation by laser sources often used for confocal microscopy, imaging, and cell sorting. NBD-labeled lipids are widely used as fluorescent analogs of native lipids in model and biological membranes to probe a wide range of processes [24,25,26,27,28,29,30,31,32]. The fluorescent NBD moiety harbors several desirable characteristics, which makes it an excellent probe for both spectroscopy and microscopy-based approaches. NBD exhibits extremely weak fluorescence in water and, when transferred to a hydrophobic medium, it fluoresces brightly in the visible range and shows a high sensitivity to its microenvironment [25,33,34,35,36,37,38]. In addition, the fluorescence lifetime of the NBD group is highly sensitive to the polarity of the surrounding environment [35,39,40,41]. NBD is moderately photostable, and NBD-labeled lipids are known to mimic endogenous lipids in studies of intracellular lipid transport [42,43,44,45,46,47,48,49].

**Figure 1:**
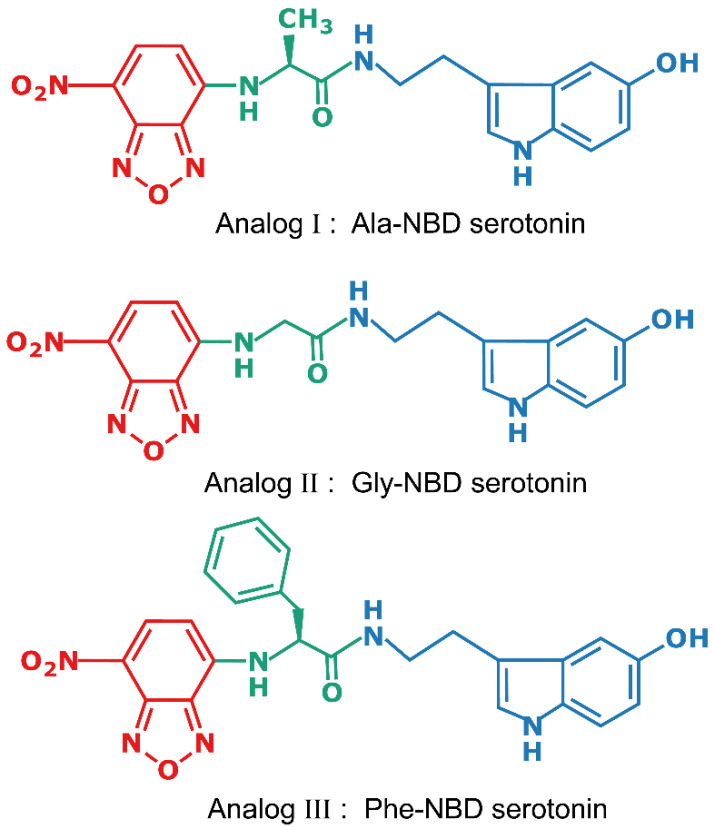
Chemical structures of the fluorescent NBD-labeled analogs of serotonin used in this study. The fluorophore (NBD) is shown in red, serotonin is shown is blue, and the linker amino acid is depicted in green.

In this paper, we report the synthesis and application of several novel fluorescent analogs of serotonin in which we have covalently attached the fluorescent NBD group to serotonin and evaluated their binding properties. The chemical structures of the fluorescent NBD-labeled analogs of serotonin are shown in Figure 1. In this work, we carried out competition binding and live cell fluorescence imaging of Chinese hamster ovary (CHO-K1) cells stably expressing the human serotonin_1A_ receptor bound to these analogs. In addition, we monitored the solvatochromic properties (i.e., the sensitivity of fluorescence parameters to the environment) of these derivatives using both spectroscopy and microscopic (spectral imaging) approaches in order to assess the usefulness of these analogs in faithfully reporting the environment of the binding site. Our results show that these novel NBD analogs of serotonin represent useful probes to monitor the localization of serotonin receptors and their interactions with ligands.

## 2. Results

### 2.1. Design of the Fluorescent Analogs of Serotonin

Agonists for serotonin receptors are chemically heterogeneous and belong to diverse chemical classes. However, a common structural feature shared by them is a basic amino group and an aromatic ring that usually has a hydroxyl (or methoxy or carbamoyl) group that could potentially form hydrogen bonds [50]. Serotonin, the natural agonist to serotonin receptors, consists of two reactive groups, the phenolic hydroxyl group and the primary amine group. Molecular modeling studies showed that the hydroxyl group is directly involved in binding to serotonin receptors by interacting with serine and threonine residues present in some of the transmembrane helices [50,51,52]. In addition, it was previously reported that the serotonin_1A_ receptor favors ligands with a hydrogen bond acceptor in a position corresponding to the hydroxyl group in serotonin [53,54]. We therefore decided to covalently attach the fluorescent NBD group to serotonin with the reactive amine group without modifying the hydroxyl group in order to not influence the binding properties (see Figure S1). The NBD group is covalently attached to serotonin through an amino acid derived short spacer arm to impart a degree of flexibility to the fluorescent analogs for efficient interaction between the ligand and the receptor without any hindrance due to steric constraints (Figure 1). It should be noted here that the primary amine group of serotonin is important for binding of serotonin to the sertotonin_1A_ receptor [55]. In this context, the chemical transformation of a basic primary amine into an amide deserves comment. Recent studies have shown that low basicity ligands for serotonin receptors, including the serotonin_1A_ receptor, do exist [56,57], and this validates the design of our NBD-labeled probes using the reactive amine group. This could account for some loss in affinity of these ligands for the serotonin_1A_ receptor (see later).

### 2.2. Competition Binding of the Fluorescent Ligands to Serotonin_1A_ Receptors

**Figure 2:**
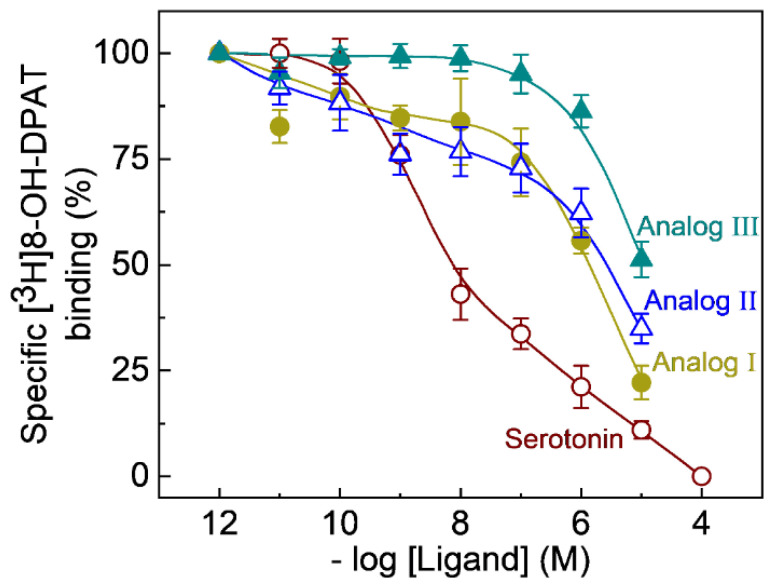
Competition binding of fluorescent NBD-labeled serotonin ligands to the human serotonin_1A_ receptor stably expressed in CHO-K1 cells. Radioligand binding assays were carried out with [^3^H]8-OH-DPAT in the presence of a range of concentrations (from 10^−12^ to 10^−4^ M) for serotonin (**○**) and NBD-labeled analogs I (●), II (Δ) and III (▲). Values of specific binding measured in the presence of fluorescent analogs are expressed as a percentage of total binding obtained at the lowest concentration of the competing ligand. The curves represent nonlinear regression fits to the experimental as described in the Supplementary Material. Data shown are means ± SE from five independent experiments. Nonspecific binding was determined in the presence of 10^−4^ M unlabeled serotonin in each case. K_i_ values were determined according to Cheng and Prusoff [60] and the average values are listed in Table 1. See Materials and Methods for other details.

Among the subtypes of serotonin receptors, the G protein-coupled serotonin_1A_ receptor is the most well-studied receptor for various reasons [15,19,20,21]. One of the major reasons is the early availability of a ligand (8-OH-DPAT) with high selectivity that permits thorough pharmacological, biochemical, and physiological characterization of the serotonin_1A_ receptor [58,59]. The interactions of the fluorescent analogs of serotonin with serotonin_1A_ receptors stably expressed in CHO-K1 cells were determined by assessing their ability to compete with the radiolabeled [^3^H]8-OH-DPAT (a specific agonist for the serotonin_1A_ receptor) for binding to the receptor. The affinities of the fluorescent ligands were determined by competition binding experiments using the NBD-labeled serotonin analogs (I-III, see Figure 1). The displacement curves of [^3^H]8-OH-DPAT by various fluorescent ligands are shown in Figure 2. We used unlabeled serotonin as a control in these experiments. Figure 2 shows that the fluorescent ligands were able to competitively inhibit the labeled agonist [^3^H]8-OH-DPAT and exhibited characteristic displacement patterns. The inhibition constant (Ki) and half maximal inhibition concentrations (IC_50_) values for the NBD-labeled fluorescent ligands are shown in Table 1. While analogs I and II show similar displacement patterns (as judged by similarity in their IC_50_ and K_i_ values), analog III shows somewhat less affinity, probably due to the presence of the bulky phenyl group. In general, analog I appeared to be the most potent analog, as apparent from competition binding assays (See Figure 2 and Table 1). The methyl group in analog I could provide enough hydrophobicity for efficient partitioning into the membrane where the binding site is located (see below).

### 2.3. Fluorescence Characteristics of the Analogs

**Figure 3:**
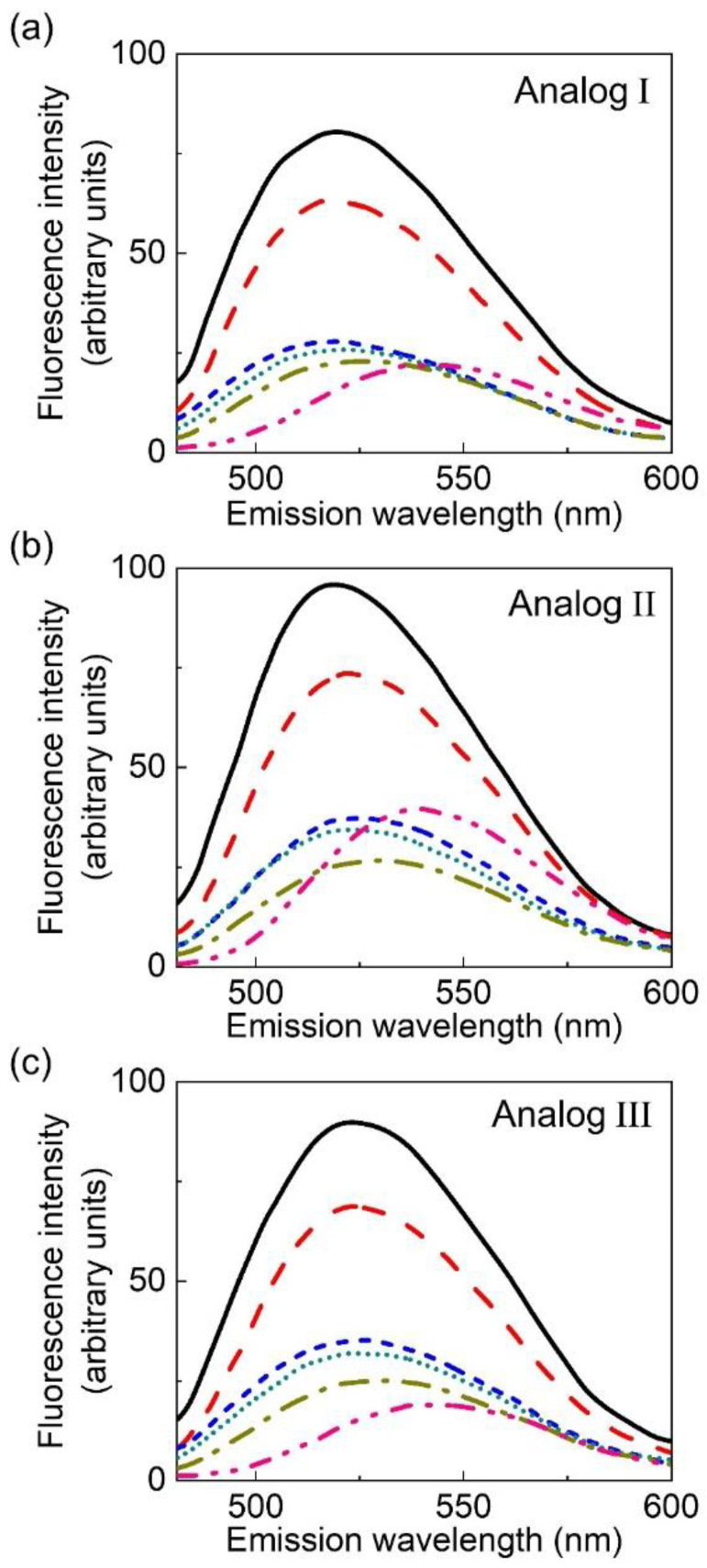
Fluorescence emission spectra of NBD-labeled analogs of serotonin (I–III) in solvents of varying polarity shown in panels (**a**–**c**), respectively. The solvents used were tetrahydrofuran (**—**), acetone (**——**), isopropanol (**---**), ethanol (**......**), methanol (**—-—**), and dimethyl sulfoxide (**—- -—**). The concentration of the NBD analogs of serotonin used was 8 μM and the excitation wavelength used was 465 nm in all cases. See Materials and Methods for other details.

The fluorescence emission spectra of NBD-labeled analogs of serotonin in various solvents are shown in Figure 3. The serotonin binding site in serotonin receptors is located inside the core of the transmembrane region where the microenvironmental polarity experienced by the ligand would be significantly lower relative to that in the bulk aqueous phase [50,52,53,54,55,62]. In order to assess the usefulness of the fluorescent ligands in such an environment, solvents were chosen, which are less polar than water (see Table 2). The fluorescence characteristics of NBD-labeled serotonin analogs in solvents of lower polarity are shown in Figure 3 and Table 2. The data show that in solvents of very low polarity (such as tetrahydrofuran), the fluorescent ligands show a significant enhancement of fluorescence. This enhancement of fluorescence intensity was accompanied by a concomitant blue shift of the emission maximum. Taken together, our results show that the environmental sensitivity displayed by the NBD-labeled serotonin analogs is in sharp contrast to the intrinsic fluorescence of serotonin, which shows little environmental sensitivity [22].

### 2.4. Specific Fluorescent Labeling of Serotonin_1A_ Receptors with NBD-Labeled Serotonin Analog

**Figure 4:**
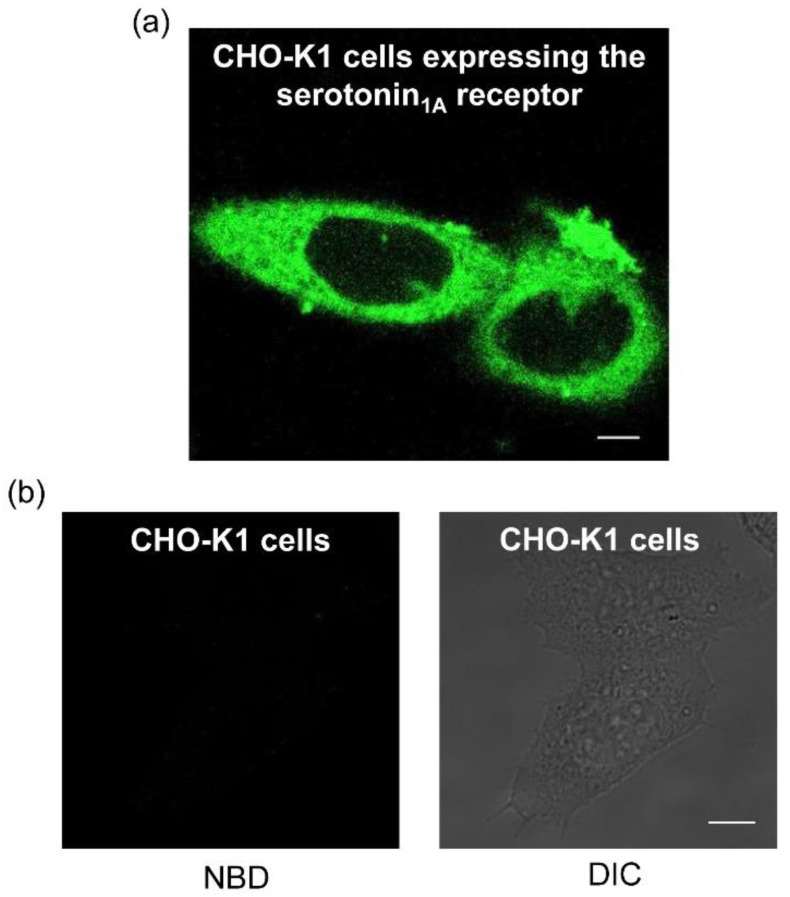
(**a**) Confocal microscopic images obtained by specific labeling of the serotonin_1A_ receptor stably expressed in CHO-K1 cells with the NBD-labeled analog of serotonin (I). Cells were grown on Lab-Tek chambers and labeled with 7 μM of analog I in HEPES-HANKS buffer, pH 7.2. NBD was excited using a 488 nm argon laser and emission was collected between 505–600 nm. Panel (**b**) shows background fluorescence of untransfected CHO-K1 cells (lacking the serotonin_1A_ receptor) labeled with 7 μM of analog I under the same conditions as in panel (**a**). The right panel shows DIC image. Scale bars represent 10 μm. See Materials and Methods for other details.

**Figure 5:**
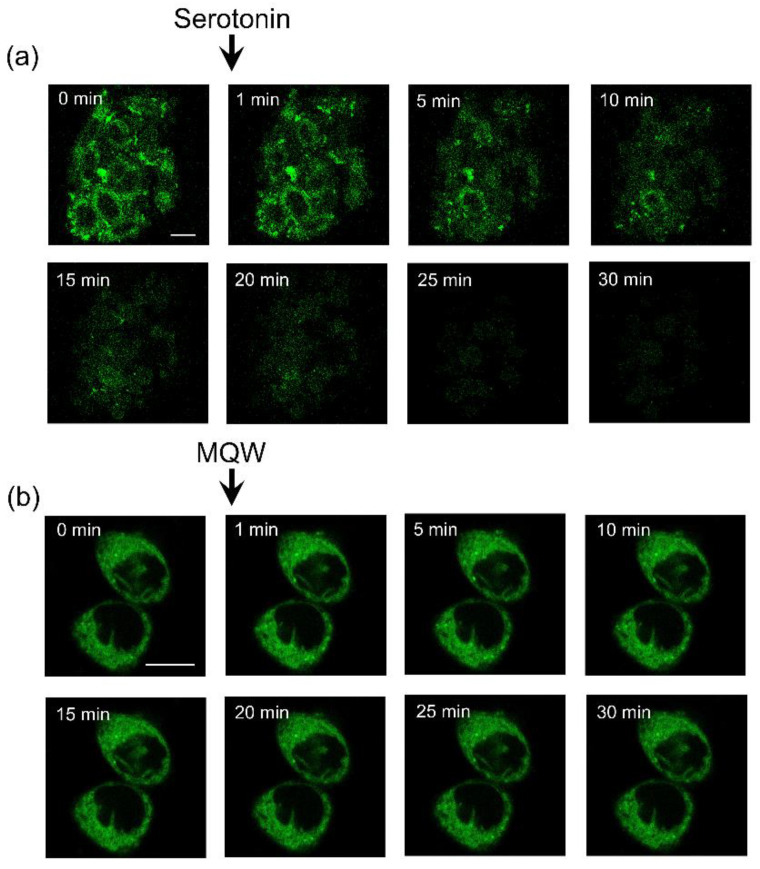
(**a**) Confocal microscopic images of the human serotonin_1A_ receptor stably expressed in CHO-K1 cells labeled with the NBD-labeled analog of serotonin (I) and chased with unlabeled serotonin. Images represent mid-plane confocal sections of the same group of cells before and after addition of serotonin. Cells were initially labeled with 7 μM of analog I in HEPES-HANKS buffer, pH 7.2 and subsequently serotonin (final concentration 7 mM) was added at 1 min time point (indicated by an arrow) from a concentrated aqueous stock solution. (**b**) Control experiment showing confocal microscopic images of the serotonin_1A_ receptor stably expressed in CHO-K1 cells labeled with the NBD-labeled analog of serotonin (I) and chased with same volume of Milli-Q water (MQW) as in panel (**a**). Images represent mid-plane confocal sections of the same group of cells before and after addition of MQW. Scale bars represent 10 μm. See Materials and Methods for other details.

The NBD-labeled serotonin analog I was used to further explore specific labeling of CHO-K1 cells heterologously expressing the human serotonin_1A_ receptor. Importantly, we earlier reported that the human serotonin_1A_ receptor heterologously expressed in these cells preserves functional characteristics of the native receptor (such as ligand binding and G-protein coupling [64]). A representative confocal micrograph obtained by labeling of human serotonin_1A_ receptors expressed in CHO-K1 cells with the NBD-labeled serotonin analog I is shown in Figure 4a. Figure 4b shows untransfected CHO-K1 cells display no labeling when incubated under identical conditions as in transfected cells. These results suggest that the nonspecific labeling of the analog was negligible. Importantly, labeling by the NBD-serotonin analog I was competed out in cells incubated with the same concentration of fluorescent ligand (as in Figure 4a) in the presence of an excess of unlabeled serotonin (see Figure 5a). The figure shows that the fluorescent signal from NBD was lost within ~10 min of incubation with unlabeled serotonin. In a control experiment, we imaged cells labeled with NBD-serotonin analog I in the presence of an equal volume of Milli-Q water to rule out any photobleaching related artifacts that could arise due to prolonged imaging (Figure 5b). Taken together, these results suggest the specific nature of labeling by the NBD-serotonin analog I in CHO-K1 cells.

### 2.5. NBD Group Senses Slow Solvent Relaxation around the Ligand Binding Pocket in the Serotonin_1A_ Receptor

Previous work from our laboratory and recent evidence from crystallographic data on the serotonin_1A_ receptor suggest that the serotonin binding pocket is localized in the interfacial region of the membrane [52,55]. Interestingly, many fluorescent molecules localized at the interfacial region of the membrane show red edge excitation shift (REES) due to the unique physicochemical properties of this region [65,66]. REES is a sensitive approach that could be utilized for monitoring the environment and dynamics around a fluorophore in complex biological systems [65,66,67,68,69]. REES is predominantly observed when polar fluorophores are present in motionally constrained media such as viscous solutions or condensed phases (e.g., proteins and membrane) in which the solvent dipolar relaxation time around a fluorophore is comparable to or longer than its fluorescence lifetime. In experimental terms, REES is defined as the shift in the wavelength of fluorescence emission maximum toward higher wavelengths due to a shift in the excitation wavelength toward the red edge of the absorption band. This arises as a result of slower rates of solvent reorientation (relaxation) in the immediate vicinity of an excited state fluorophore, which is dependent on motional restriction imposed on the solvent molecules around the fluorophore. As a result, REES can be effectively used to monitor the organization and dynamics of the environment (which is represented by the relaxing solvent (water) molecules), using the fluorophore merely as a reporter group. Although the bulk of REES analysis has used spectroscopic (cuvette-based) measurements, we utilized confocal microscopy based high resolution spectral imaging to monitor the REES of the NBD analog I bound to serotonin_1A_ receptor in live CHO-K1 cells.

**Figure 6:**
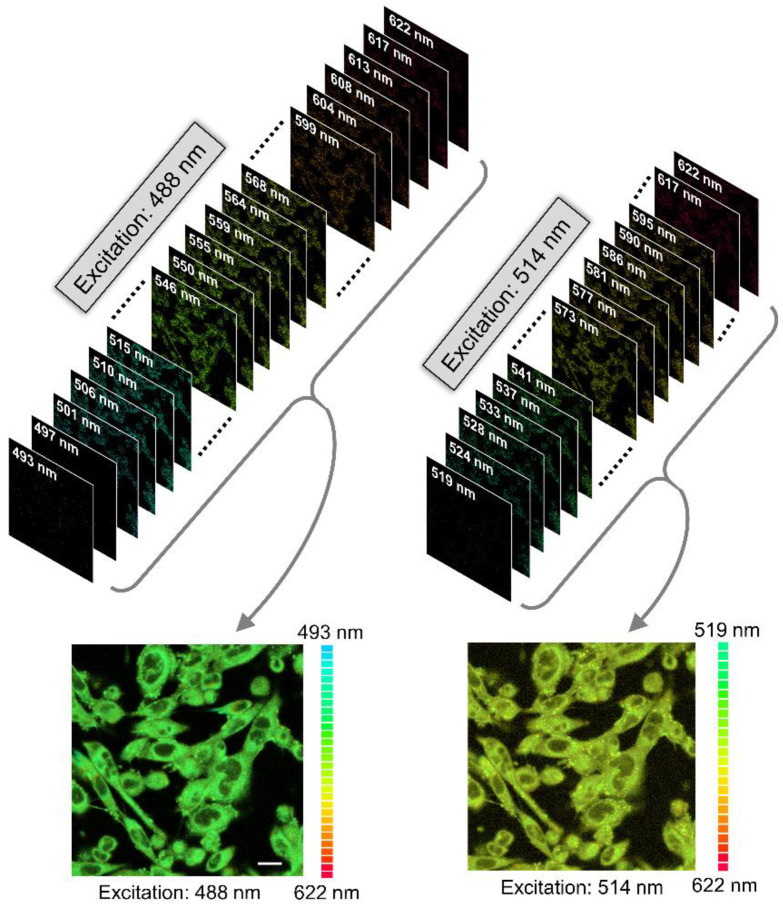
Spectral image stacks consisting of a collection of images showing serotonin_1A_ receptor labeled using NBD-serotonin analog I, each of which measured at a specific wavelength (highlighted on top left corner of image) for two different excitation wavelengths. Unlike a typical confocal image, which is acquired over a wavelength range of the detector, a spectral image contains a collection of images of the same field captured at different wavelengths. As a result, spectral images provide a complete emission spectrum of the fluorophore at every pixel location. The corresponding overlaid image of the linearly unmixed spectral image stack is shown at the bottom. The individual images in the stack were colored according to the color-map showed beside the overlaid image. Note that the overall red-shifted emission of the NBD-serotonin analog I when excited at the red edge (514 nm) indicates REES. The scale bar represents 10 μm. See Materials and Methods for other details.

**Figure 7:**
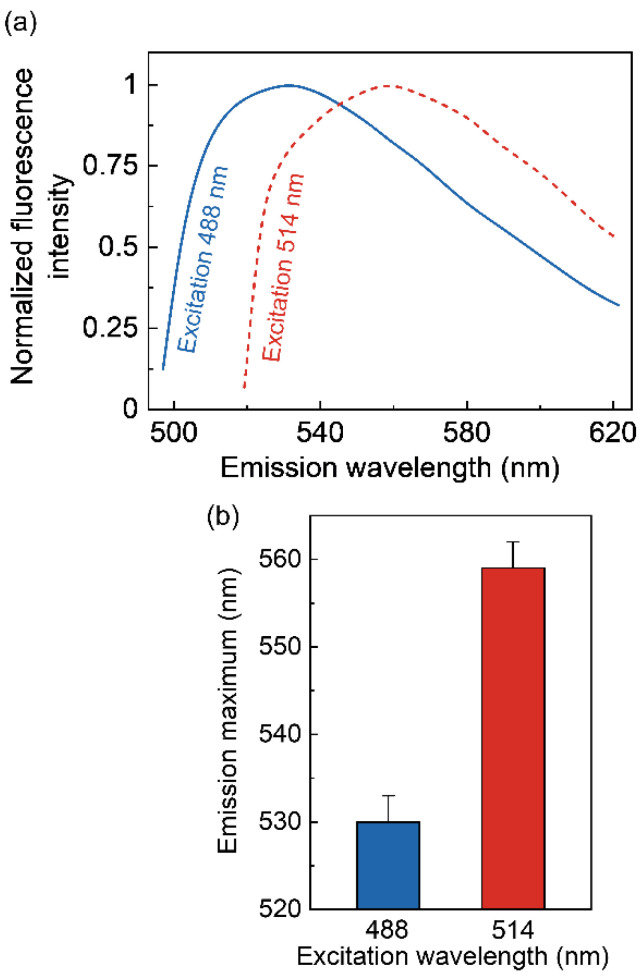
(**a**) Representative fluorescence emission spectra of NBD-serotonin analog I bound to the serotonin_1A_ receptor expressed in CHO-K1 cells at different excitation wavelengths. The excitation wavelengths were 488 (—) and 514 (- - -) nm. All spectra are intensity-normalized at the emission maximum. The concentration of NBD-serotonin analog I was 7 μM. A Zeiss LSM 880 inverted spectral imaging confocal microscope with a multichannel spectral detector was used to construct pixel-by-pixel fluorescence emission spectra from the confocal images. (**b**) Effect of changing excitation wavelength on the wavelength of maximum emission for NBD-serotonin analog I. Data represent means ± SE of at least 35 different fields from three independent experiments. See Materials and Methods for other details.

We utilized a confocal microscopic set-up equipped with a multichannel spectral detector to construct pixel-by-pixel fluorescence emission spectra from confocal images of live cells (see Figure 6). For this, we used two excitation lines (488 and 514 nm) of an argon laser and monitored the emission of NBD from serotonin_1A_ receptor bound NBD-serotonin analog I between 493–622 nm (for excitation at 488 nm) and 519–622 nm (for excitation at 514 nm) with an interval of ~4 nm (Figure 6). Figure 7a shows the representative fluorescence emission spectra of NBD-serotonin analog I bound to the human serotonin_1A_ receptor in cells with increasing excitation wavelength Upon excitation at 488 nm, NBD-serotonin analog I in the serotonin_1A_ receptor bound state displayed an emission maximum at 530 nm (Figure 7a,b). Interestingly, the emission maximum exhibited a shift toward longer wavelengths when the excitation wavelength was shifted to longer wavelength (Figure 7). Figure 7b shows that when NBD was excited at 514 nm, the maximum of fluorescence emission shifted to 559 nm, corresponding to a REES of 29 nm. Such a shift in the fluorescence emission maximum with a change in excitation wavelength suggests that the fluorescent NBD group in NBD-serotonin analog I is located in a motionally restricted environment.

## 3. Discussion

Fluorescence-based approaches represent a convenient way to monitor and analyze biomolecular interactions at the molecular and cellular level. The advantages of employing fluorescence techniques include sensitivity, suitable time scale, minimal perturbation (non-invasive), and the dynamic nature of the information obtained. Due to significant improvements in instrumentation, the sensitivity of detection of fluorescence has immensely improved in the past few years. With the rapid development of confocal microscopy, the study of receptor biology using fluorescently labeled ligands has become popular [70,71]. Fluorescently labeled ligands have proven to be very useful in monitoring receptor binding sites, kinetics, regulation, clustering, dynamics, and trafficking [72,73,74,75,76]. Fluorescent ligands are often more attractive than radioligands, since affinity measurements can be performed by flow cytometry and receptor dynamics can be studied both at the cellular and the molecular level. In addition, structural information on receptor-ligand interaction can be obtained from fluorescence resonance energy transfer (FRET) experiments using appropriate donor and acceptor fluorescent labels [77]. Dynamic measurements (such as fluorescence recovery after photobleaching (FRAP)) can be conveniently used to monitor the lateral mobility (diffusion) of labeled receptors in tissue samples or in a single cell [78,79,80,81,82]. Importantly, fluorescence correlation spectroscopy (FCS), often using single receptor molecule, allows monitoring the interaction between the receptor and ligand [83] or lipid [84], and has been utilized for drug screening [85].

Serotonin exerts its diverse physiological actions by binding to specific receptors present in the plasma membrane [4,14,16]. For serotonin receptors in the GPCR family (except the serotonin_3_ receptor which is an ion channel), the ligand binding site lies within the transmembrane core of the receptor [52,55,86,87,88,89]. Interestingly, the conserved residues in GPCRs are predominantly located within the hydrophobic regions and not in the extra- and intracellular loops that connect the transmembrane helices [90]. Sequence alignment of GPCRs revealed several conserved polar residues within the transmembrane segments, indicating that the hydrophilic residues in the transmembrane helices are the most probable sites of receptor-agonist interactions [90]. In the case of serotonin_1A_ receptors, mutagenesis [50,62], molecular dynamics simulations [53,54], and cryo-EM structures [55] have revealed that the site for ligand binding is located within the transmembrane core of the receptor. Since the polarity of the microenvironment experienced by serotonin in such a position would be considerably lower than the bulk aqueous phase, we reasoned that a probe whose fluorescence is sensitive to this change in polarity upon binding would be helpful. In other words, the fluorescent probe labeled to serotonin should be solvatochromic to be able to ‘sense’ and faithfully report the microenvironment of the binding site. NBD is an ideal probe for this purpose, since its fluorescence is known to be highly sensitive to the polarity of the surrounding environment where it is placed [24,25]. This is primarily attributed to the large dipole moment change (~3.9 D) of the NBD group upon excitation [38]. NBD exhibits extremely weak fluorescence in water, and when transferred to a hydrophobic medium, it fluoresces brightly in the visible range and shows a high sensitivity to its microenvironment [24,25,33,34,35,36,37,38]. In addition, the fluorescence lifetime of the NBD group is highly sensitive to the polarity of the surrounding environment [35,39,40,41].

NBD-labeled ligands have previously been used to label the nicotinic acetylcholine receptor [91], adenosine receptors [92], dopamine receptors [93], opioid receptors [94], and benzodiazepine receptors [95]. In the case of serotonin_3_ receptors, photolabile derivatives of serotonin have been developed for kinetic investigations [96]. In this work, we report the synthesis and application of NBD-labeled serotonin analogs. In these analogs, the NBD group is covalently attached to serotonin in such a manner that the binding properties do not exhibit very large change, as apparent from the apparent dissociation constants of the fluorescent analogs shown in Table 1. We showed that the fluorescent ligands competitively displace the serotonin_1A_ receptor specific radiolabeled agonist [^3^H]8-OH-DPAT from the receptor, thereby demonstrating their binding specificity to the serotonin receptors. We further showed that serotonin_1A_ receptors expressed in CHO-K1 cells could be specifically labeled with one of the fluorescent ligands with minimal nonspecific labeling. Importantly, a useful feature of these fluorescent ligands is the environmental sensitivity of their fluorescence, as evident from our results (Table 2, Figure 3 and Figure 7). This could prove to be helpful in exploring the physicochemical properties of the molecular environment (such as polarity) of the serotonin binding site in future studies.

## 4. Materials and Methods

### 4.1. Materials

Carbobenzyloxy-amino acids (alanine, glycine, and phenylalanine), EDTA, gentamicin sulfate, HEPES, MgCl_2_, MnCl_2_, penicillin, polyethylenimine, serotonin hydrochloride, streptomycin sulfate, Tris, and trypsin were obtained from Sigma Chemical Co. (St. Louis, MO, USA). NBD chloride was purchased from Molecular Probes (Eugene, OR, USA). DMEM/F-12 (Ham’s nutrient mixture, 1:1) and fetal calf serum (FCS) were obtained from Life Technologies Invitrogen/Life Technologies (Grand Island, NY, USA). [^3^H]8-OH-DPAT (127.2 Ci/mmol) was obtained from DuPont New England Nuclear (Boston, MA, USA). GF/B glass microfiber filters were obtained from Whatman International (Kent, UK). The BCA reagent kit for protein estimation was obtained from Pierce (Rockford, IL, USA). Solvents used were of spectroscopic grade. The purity of the solvents was checked by the *E*_T_(30) procedure (see below). The *E*_T_(30) dye was a kind gift from Dr. Christian Reichardt (Philipps University, Marburg, Germany). All other chemicals used were of the highest available purity. Water was purified through a Millipore Milli-Q system and used throughout.

### 4.2. Synthesis of NBD-Labeled Serotonin Analogs

Synthesis of the NBD-labeled serotonin analogs were carried out as described in Supplementary Material (Section S1).

### 4.3. Cell Culture

CHO-K1 cells were maintained in DMEM/F-12 medium supplemented with 2.4 g/L sodium bicarbonate, 10% (*v*/*v*) FCS, 60 μg/mL penicillin, 50 μg/mL streptomycin and 50 μg/mL gentamycin sulfate (complete DMEM/F-12) in a humidified atmosphere with 5% CO_2_ at 37 °C. CHO-K1 cells heterologously expressing the human serotonin_1A_ receptor were maintained in complete DMEM/F-12 medium supplemented with 0.2 mg/mL G418 in a humidified atmosphere with 5% CO_2_ at 37 °C. Cells were harvested after reaching ~70–80% confluency (~3 days).

### 4.4. Cell Membrane Preparation

Confluent cells were washed once with phosphate buffered saline (PBS) and scraped off in ice-cold hypotonic buffer (10 mM Tris, 5 mM EDTA, pH 7.4) using a cell scraper. The cell suspension obtained was homogenized in a Bellco homogenizer (maximum speed for 2 min) at 4 °C. The homogenate obtained was centrifuged at 300,000× *g* for 10 min in a Beckman ultracentrifuge. The supernatant was discarded and the pellet was resuspended in buffer A (50 mM Tris, pH 7.4), stored at −70 °C, and used for radioligand binding assays. Protein concentration was determined using BCA reagent [97].

### 4.5. Radioligand Binding Assay

Radioligand binding assays in isolated membranes were carried out as described previously [64], with some modifications. See Supplementary Material (Section S2) for more details.

### 4.6. Saturation Binding Assay

Saturation binding assays were carried out as described previously [64]. Scatchard plots (i.e., plots of RL*/L* vs. RL* where L* is the total ligand concentration) were analyzed. The dissociation constants (K_d_) were obtained from the negative inverse of the slopes, determined by linear regression analysis of the plots (r = 0.90–0.99). See Supplementary Material (Section S3) for more details.

### 4.7. Competition Binding Assay

Competition binding assays were carried out as described previously [64], with some modifications. The final concentrations of the competitive ligands in the assay tubes ranged from 10^−12^ to 10^−4^ M. The average of the K_i_ values for the competitive ligands are shown in Table 1. See Supplementary Material (Section S4) for more details.

### 4.8. Checking the Purity of Organic Solvents Using the E_T_(30) Dye

The purity of organic solvents were checked using the *E*_T_(30) dye as described previously [38]. The *E*_T_(30) values obtained by us showed a maximum deviation of <0.6% from the reported values for the solvents used in this study. See Supplementary Material (Section S5) for more details.

### 4.9. Steady State Fluorescence Measurements

Steady state fluorescence measurements were performed with a Hitachi F-4010 spectrofluorometer (Tokyo, Japan) using 1 cm path length quartz cuvettes. Excitation and emission slits with a bandpass of 5 nm were used for all measurements. Background intensities of samples in which fluorophores were omitted were negligible in most cases and were subtracted from each sample spectrum to cancel out any contribution due to the solvent Raman peak and other scattering artifacts. Data shown are representative of three independent measurements, and the reported emission maxima in Table 2 in each case were identical (or ±1 nm of the values reported). The concentration of NBD-labeled serotonin analogs was calculated from their molar extinction coefficient of 20,000 M^−1^ cm^−1^ at 460 nm [24]. The excitation wavelength used was 465 nm for all measurements. For fluorescence measurements, samples were prepared by drying NBD-labeled serotonin analogs (in methanol) under a stream of nitrogen while warming gently (~40 °C). After further drying under a high vacuum for at least 12 h, 1.5 mL of solvent was added to the dried film and vortexed for 3 min to dissolve the NBD-labeled serotonin analogs in the solvent. The concentration of the fluorescent analogs was 8 μM in all cases. The solvents used were tetrahydrofuran, acetone, isopropanol, ethanol, methanol, and dimethyl sulfoxide.

### 4.10. Absorption Measurements

Absorption spectra were recorded using a Hitachi U-2000 UV-visible absorption spectrophotometer (Tokyo, Japan) after appropriate baseline corrections. Quartz cuvettes with a path length of 1 cm were used.

### 4.11. Fluorescent Labeling of CHO-K1 Cells Stably Expressing Serotonin_1A_ Receptors

CHO-K1 cells stably expressing the human serotonin_1A_ receptor were used for labeling with the NBD-labeled serotonin analog (analog I). Cells were plated on Lab-Tek chamber slides (Nunc, Denmark) and allowed to grow for 2 days. The stock solution of NBD-labeled serotonin analog I was prepared in methanol. The chamber slides were washed four times with HEPES-HANKS (10 mM, pH 7.2) buffer before labeling. Analog I (final concentration 7 μM) in HEPES-HANKS buffer was then added to the chamber slides and incubated at 37 °C for 30 min (the final methanol concentration was 0.2% (*v*/*v*)). The chamber slides were then washed three times with HEPES-HANKS buffer to remove unbound analog I, and confocal imaging was carried out at room temperature (~23 °C) in HEPES-HANKS buffer. In a control experiment, untransfected CHO-K1 cells (without expressing the serotonin_1A_ receptor) were labeled with analog I (7 μM) in HEPES-HANKS buffer for 30 min at 37 °C. In another experiment, CHO-K1 cells stably expressing the human serotonin_1A_ receptor were first labeled with analog I and subsequently incubated with 7 mM unlabeled serotonin for various time points.

### 4.12. Fluorescence Microscopy and Imaging

Images were acquired using an inverted Zeiss LSM 880 confocal microscope (Jena, Germany) with a 63×/1.4 NA oil immersion objective under one airy condition. NBD was excited using the 488 nm line of an Argon laser, and emission was collected from 500–600 nm. For time series experiments, images were collected at an interval of 1 min for a total duration of 30 min. Image analyses were performed using ImageJ (NIH, Bethesda, MD, USA). Spectral imaging was carried out using a 34 channel GaASP spectral detector with a spectral resolution of ~4 nm. Images were collected using 488 and 514 nm lines of an Argon laser and spectral images were collected between 493–622 nm and 519–622 nm, respectively. Spectral unmixing and processing of the obtained images were performed using the ZEN imaging software (Zeiss, Jena, Germany).

## 5. Conclusions

Taken together, the novel NBD-labeled serotonin analogs represent a useful class of probes and offer an attractive fluorescent approach to study serotonin receptors and their interactions with specific ligands and lipids and their cellular localization and regulation as well as to monitor the mobility and dynamics of serotonin receptors in normal and diseased states. Considering the multiple roles of serotonin receptors both in the central and peripheral nervous systems, these fluorescent ligands should prove to be useful in future studies involving serotonergic systems.

## Figures and Tables

**Table 1:** **IC_50_ and K_i_ values of fluorescent NBD-labeled serotonin analogs for binding to serotonin_1A_ receptors stably expressed in CHO-K1 cells ^a^**.

NBD-Labeled Analogs	IC_50_ (μM)	K_i_ (μM)
I	1.74 ± 0.44	1.41 ± 0.36
II	3.43 ± 1.73	3.15 ± 1.41
III	13.70 ± 3.36	11.15 ± 2.73

^a^ The IC_50_ and apparent dissociation constants (K_i_) values shown in the table represent the means ± SE of duplicate points from five independent experiments. K_i_ for the fluorescent ligands were calculated as described in the Supplementary Material [61]. See Materials and Methods for other details.

**Table 2:** **Solvent effects on the fluorescence characteristics of NBD-labeled serotonin analogs**.

Solvents	Dielectric Constant ^b^	Emission Maximum (nm)			Relative Intensity ^c^		
		Analog			Analog		
		I	II	III	I	II	III
Tetrahydrofuran	7.58	520	520	525	3.7	2.4	4.8
Isopropanol	18.30	520	524	525	1.3	0.9	1.8
Acetone	20.70	520	523	526	2.9	1.9	3.6
Ethanol	24.30	520	526	528	1.2	0.9	1.7
Methanol	32.63	526	530	532	1.0	0.6	1.3
Dimethyl sulfoxide	46.45	541	540	542	1.0	1.0	1.0

^b^ From [63]. ^c^ Calculated by measuring fluorescence intensity at the respective emission maximum upon excitation at 465 nm and normalized to the fluorescence intensity in dimethyl sulfoxide.

## Data Availability

The data presented in this study are available on request from the corresponding author.

## Supplementary Materials

Method S1: Synthesis of NBD-labeled serotonin analogs, Method S2: Radioligand binding assay, Method S3: Saturation binding assay, Method S4: Competition binding assay, Method S5, Checking the purity of organic solvents using the ET(30) dye, Figure S1: A three-step protocol followed for the synthesis of NBD-labeled serotonin analogs I-III, Figure S2: Mass spectra of NBD-Ala-serotonin analog I, Figure S3: Absorption spectra of NBD-labeled analogs of serotonin (I) in solvents of varying polarity

## Abbreviations

BCA, bicinchoninic acid; Cbz, carbobenzyloxy; DIPEA, N,N-diisopropylethylamine; DMEM, Dulbecco’s modified Eagle’s medium; DMF, N,N-Dimethylformamide; EDTA, ethylenediaminetetraacetic acid; EDCI, 1-ethyl-3-(3-dimethylaminopropyl)carbodiimide hydrochloride; ET(30), 2,6-diphenyl-4-(2,4,6-triphenyl-N-pyridinio)phenoxide; HEPES, 4-(2-hydroxyethyl)-1-piperazine ethanesulfonic acid; HOBt, 1-hydroxybenzotriazole; 8-OH-DPAT, 8-hydroxy-2(di-N-propylamino)tetralin; NBD, 7-nitrobenz-2-oxa-1,3-diazol-4-yl; REES, red edge excitation shift; Tris, tris-(hydroxymethyl)aminomethane.
