# Supplementary material for: Environment-Sensitive Fluorescence of 7-Nitrobenz-2-oxa-1,3-diazol-4-yl (NBD)-Labeled Ligands for Serotonin Receptors
Source: Molecules. 2021 Jun 24;26(13):3848. doi: 10.3390/molecules26133848 (PMC8270269; doi:10.3390/molecules26133848)
Supplement: Supplementary file 1 [file molecules-26-03848-s001.zip › molecules-1210502-supplementary.pdf]

Supplementary Data

Environment-sensitive Fluorescence of 7-Nitrobenz-2-oxa-1,3-diazol-4-yl (NBD)-labeled Ligands for Serotonin Receptors

Parijat Sarkar[1], Kaleeckal G. Harikumar[1,3], Satinder S. Rawat[1,4], Sanjib Das[2], Tushar K. Chakraborty[2,5] and Amitabha Chattopadhyay[1,*]

[1]CSIR-Centre for Cellular & Molecular Biology, Uppal Road, Hyderabad 500 007, India
[2]CSIR-Indian Institute of Chemical Technology, Uppal Road, Hyderabad 500 007, India

[3]Present address: Department of Molecular Pharmacology and Experimental Therapeutics, Mayo Clinic, Scottsdale, AZ 85259, USA

[4]Present address: Taysha Gene Therapies, 3000 Pegasus Park Drive, Suite 1430 Dallas, Texas 75247, USA

[5]Present address: Department of Organic Chemistry, Indian Institute of Science, Bengaluru 560 012, India

*Address correspondence to Amitabha Chattopadhyay,
Tel: +91-40-2719-2578; E-mail: amit@ccmb.res.in

S1. Synthesis of NBD-labeled Serotonin Analogs

All reactions were carried out in oven or flame-dried glassware with magnetic stirring under nitrogen atmosphere using dry, freshly distilled solvents unless otherwise mentioned. DMF was purified by azeotroping twice with equal volumes of dry benzene, followed by distillation over ninhydrin under reduced pressure. Fractions with boiling point of 76 °C (at 39 mm of Hg pressure) were collected. Dichloromethane (CH_2Cl_2) was purified by distilling over powdered CaH_2 and stored over 4 Å molecular sieves.

The progress of reactions was monitored by thin layer chromatography carried out on 0.25 mm silica gel (60F-254) plates (E. Merck, Darmstadt, Germany) with UV light, iodine, 7% ethanolic phosphomolybdic acid-heat and 2.5% ethanolic anisaldehyde (with 1% acetic acid and 3.3% concentrated sulfuric acid)-heat as developing agents. Silica gel finer than 200 mesh (Acme, India) was used for flash column chromatography. Yields refer to chromatographically and spectroscopically homogeneous materials unless otherwise stated. Reaction products were characterized by spectroscopic methods.

In a typical experiment, carbobenzyloxy-alanine (135 mg, 0.61 mmol) in CH_2Cl_2 (5 ml) was treated sequentially with HOBt (82.3 mg, 0.61 mmol) and EDCI (117 mg, 0.61 mmol) at 0 °C and the reaction mixture was stirred at the same temperature for 30 minutes. A solution of serotonin hydrochloride (128.6 mg, 0.61 mmol) in DMF (0.5 ml), neutralized with triethylamine (0.12 ml, 0.9 mmol), was added to the above reaction mixture. After stirring for 2 hour at room temperature (~25 °C), the reaction mixture was diluted with ethyl acetate (20 ml), washed with saturated ammonium chloride (10 ml), $NaHCO_3$ (10 ml), brine (10 ml), dried over Na_2SO_4, and concentrated in vacuum. Column chromatography (SiO_2, 1-5% methanol in chloroform as eluant) gave pure amide IV in 89% yield (207 mg) as a white solid. A solution of IV (72 mg, 0.19 mmol) in methanol (1 ml) was hydrogenated under atmospheric pressure using Pd on activated carbon (10%, 15 mg) for 30 min. The reaction mixture was then filtered through a short pad of Celite and the filter cake was washed with methanol (2x2 ml). The combined filtrate and washings were concentrated *in vacuo* to get the deprotected amine VII in quantitative yield (46 mg) as a white solid. The amine

VII dissolved in ethanol (1 ml) was treated sequentially with DIPEA (0.05 ml, 0.28 mmol) and NBD chloride (41.5 mg, 0.2 mmol) at room temperature. After stirring for 12 hours, the reaction mixture was concentrated *in vacuo* and subjected to silica gel column chromatography (SiO_2, 1-5% methanol in chloroform eluant) to get the pure NBD-labeled serotonin analog I in 22% yield (17 mg) as an orange-red solid. Other analogs (II and III) were synthesized following similar procedure as described above (see Figure S1). High resolution MS of the Ala-NBD serotonin analog I is shown in Fig. S2.

S2. Radioligand Binding Assay

For radioligand binding assays, tubes in duplicate containing 50-100 µg of membrane protein were mixed with 0.29 nM [^{3}H]8-OH-DPAT in a total volume of 1 ml of buffer B (50 mM Tris, 1 mM EDTA, 10 mM $MgCl_2$, 5 mM $MnCl_2$, pH 7.4) and were incubated for 1 hour at room temperature (~23 °C). Nonspecific binding was determined by performing the assay in presence of 10 µM unlabeled serotonin. The incubation was terminated by rapid filtration under vacuum in a Millipore multiport filtration apparatus through Whatman GF/B (1.0 µm pore size) 2.5 cm diameter glass microfiber filters which were presoaked in 0.3% (w/v) polyethylenimine for 3 hours [1]. The filters were washed three times with 3 ml of cold water (4 °C), dried, and the retained radioactivity was measured in a Packard Tri-Carb 1500 scintillation counter (PerkinElmer, Waltham, MA) using 5 ml of scintillation fluid.

S3. Saturation Binding Assay

Saturation binding assays were carried out using varying concentrations of the agonist [^{3}H]8-OH-DPAT (0.1-7.5 nM). Nonspecific binding was measured in presence of 10 µM unlabeled serotonin. Binding assays were carried out at room temperature (~23 °C) as mentioned above. Binding data were analyzed as described earlier [2]. The concentration of bound ligand (RL^*) was calculated from the equation:

$$RL^* = 10^{-9} \times B/(V \times SA \times 2220)\ M \qquad (S1)$$

where B = bound radioactivity in dpm (*i.e.*, (total dpm - nonspecific dpm)), V is the assay volume in ml, and SA is the specific activity of the radioligand.

S4. Competition Binding Assay

For competition binding assays, tubes in duplicate containing 50-100 µg of membrane protein were incubated in presence of 0.29 nM [^{3}H]8-OH-DPAT in a total volume of 1 ml of buffer B. Nonspecific binding was determined by performing the assay in the presence of 10^{-4} M of unlabeled serotonin. Radioligand binding assay was carried out at room temperature (~23 °C) for 1 hour as mentioned above. The concentrations of the competitive ligand leading to half-maximal inhibition of specific agonist binding (IC_{50}) were calculated by nonlinear regression fitting of the data to a four-parameter logistic function:

$$B = a[1+(x/I)^s]^{-1} + b \qquad (S2)$$

where, B is the binding of specific agonist in presence of the competitive ligand normalized to binding observed in absence of the competitive ligand, x denotes concentration of the competitive ligand, a is the range (y_{max} - y_{min}) of the fitted curve on the ordinate (y-axis), I is the IC_{50} concentration, b is the background of the fitted curve (y_{min}) and s is the slope factor. The affinity values of the displacing ligands are expressed as the apparent dissociation constants (K_i) for the fluorescent ligands, where K_i is calculated from IC_{50} value using the Cheng-Prusoff equation [3]:

$$K_i = IC_{50} / (1+[L]/K_d) \text{ M} \qquad (S3)$$

where IC_{50} is the concentration of the fluorescent ligand leading to 50% inhibition of specific binding and [L] and K_d are the concentration and dissociation constant of the labeled ligand, respectively. K_d value for [^{3}H]8-OH-DPAT binding to the serotonin$_{1A}$ receptor stably expressed in CHO-K1 cells was found to be 1.27 nM.

S5. Checking the Purity of Organic Solvents using the E_T(30) Dye

The E_T(30) dye is a pyridinium-*N*-phenoxide betaine dye which exhibits a negatively solvatochromic π –> π* absorption band with an intramolecular charge transfer character. This

molecule undergoes one of the largest known solvent-induced shifts in absorption maximum, amounting to 357 nm (9730 cm^{-1}) in going from water (453 nm) to diphenyl ether (810 nm). This extremely large solvent-induced shift of the visible $\pi \rightarrow \pi^*$ absorption band has been used to introduce an empirical parameter of solvent polarity, called the $E_T(30)$ value [4]. According to the following equation, the $E_T(30)$ value for a solvent is defined as the transition energy of the dissolved betaine dye measured in kcal/mol:

$$E_T(30) = hc\bar{n}\,N_A = 2.859 \times 10^{-3}\,\bar{n} \qquad \text{(S4)}$$

where h is Planck's constant, c is the velocity of light, $\bar{n}$ is the wavenumber (in cm^{-1}) of the photon which produces the electronic transition, and N_A is Avogadro's number. Due to extremely large solvatochromism of this dye, $E_T(30)$ values provide an excellent characterization of the polarity of solvents and therefore serve as sensitive indicators for trace amounts of water or any other contaminant present in any solvent [5]. $E_T(30)$ values have been previously determined for more than 270 pure solvents [4].

For estimating the purity of solvents used, a few grains of $E_T(30)$ dye were dissolved in the solvent of interest, and its absorption maximum was monitored. From this absorption maximum, the $E_T(30)$ value was calculated using Eq. (S4). The $E_T(30)$ value so obtained was then compared with the values reported in the literature [4].

Figure S1
Sarkar *et al.*

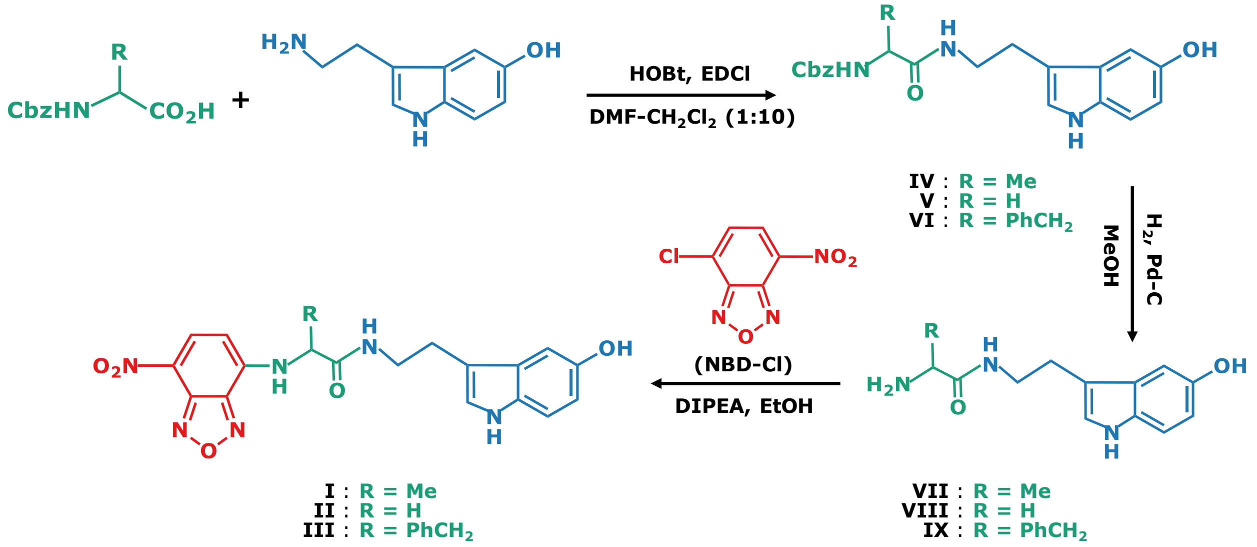

Figure S1. A three-step protocol followed for the synthesis of NBD-labeled serotonin analogs I-III (I: NBD-alanine-5-hydroxytryptamide; II: NBD-glycine-5-hydroxytryptamide; III: NBD-phenylalanine-5-hydroxytryptamide). The first step involved coupling of serotonin with the corresponding *N*-Cbz-protected amino acids by solution phase peptide coupling method using EDCI and HOBt as coupling agents and amine-free dry DMF-CH_2Cl_2 (1:10, v/v) as solvent. The resulting amides IV-VI, obtained in about 85-90% isolated yields, were then subjected to deprotection by hydrogenation using 10% Pd on activated carbon as catalyst to get quantitative yields of the free amines VII-IX. In the last step, the free amines were reacted with NBD chloride in the presence of DIPEA in ethanol. The products I-III, thus formed in about 20-25% yields, were purified by silica gel column chromatography.

Figure S2
Sarkar et al.

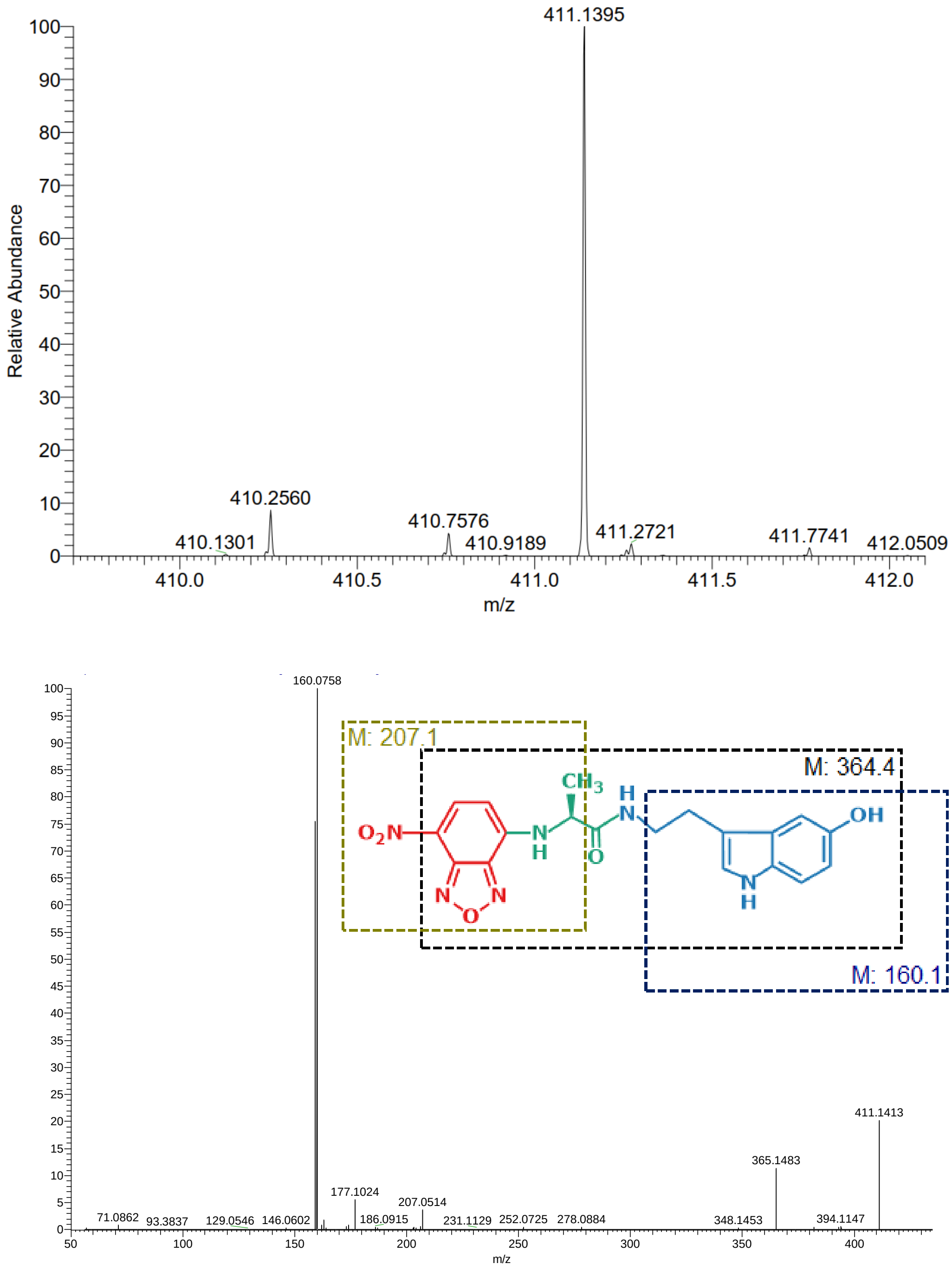

Figure S2. Mass spectra of NBD-Ala-serotonin analog I show mass of 411.14 Da (upper panel). Fragments obtained after tandem MS are indicated with dotted box in the NBD-Ala-serotonin analog I structure (bottom panel).

Figure S3
Sarkar et al.

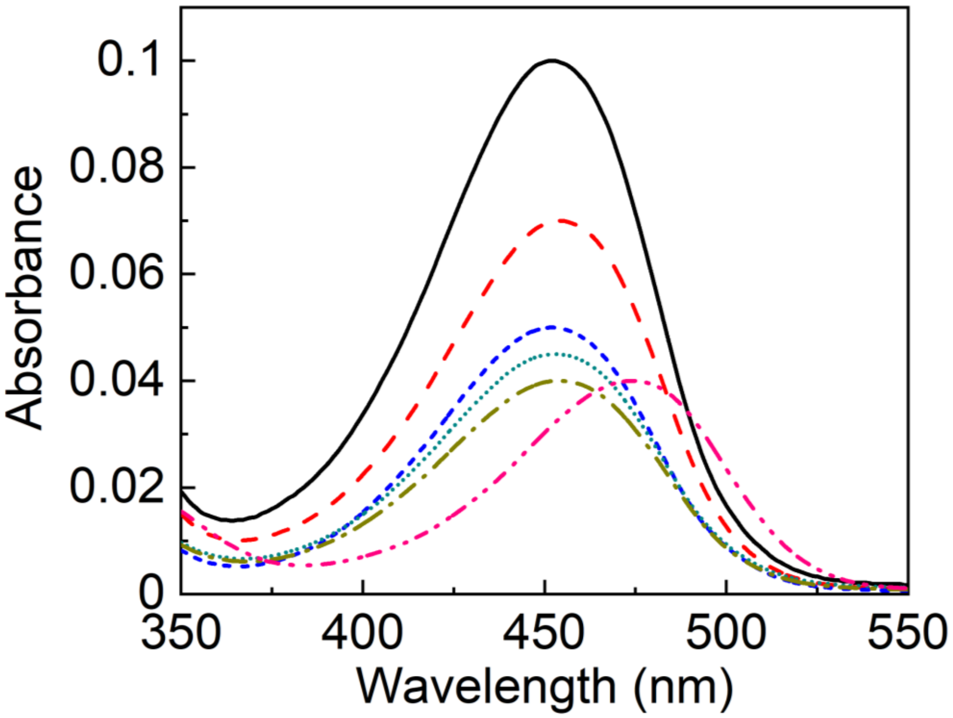

Figure S3. Absorption spectra of NBD-labeled analogs of serotonin (I) in solvents of varying polarity. The solvents used were tetrahydrofuran (—), acetone (— —), isopropanol (---), ethanol (......), methanol (— - —) and dimethyl sulfoxide (— - - —). The concentration of the NBD analogs of serotonin used was 2 μM. See Materials and Methods for other details.

References

1. Bruns, R.F.; Lawson-Wendling, K.; Pugsley, T.A. A rapid filtration assay for soluble receptors using polyethylenimine-treated filters. *Anal. Biochem.* **1983**, *132*, 74-81.
2. Hulme, E.C. *Receptor-Effector Coupling: A Practical Approach*. IRL Press, New York, 1990.
3. Cheng, Y.; Prusoff, W.H. Relationship between the inhibition constant (K_i) and the concentration of inhibitor which causes 50 per cent inhibition (IC_{50}) of an enzymatic reaction. *Biochem. Pharmacol.* **1973**, *22*, 3099-3108.
4. Reichardt, C. *Solvents and Solvent Effects in Organic Chemistry*. VCH Publishers, Weinheim, Germany, 1988.
5. Mukherjee, S.; Chattopadhyay, A.; Samanta, A.; Soujanya, T. Dipole moment change of NBD group upon excitation studied using solvatochromic and quantum chemical approaches: Implications in membrane research. *J. Phys. Chem.* **1994**, *98*, 2809-2812.
